# Tomatoes and Melons

**Published:** 1858-09

**Authors:** 


### TOMATOES AND MELONS.

USE tomatoes largely, both at breakfast and dinner; take them hot or cold, cooked or raw, with vinegar, or without vinegar, fried in sugar and butter, or stewed, with salt and pepper. Their healthful properties consist in their being nutritious, easily digested, and promotive of that daily regular action of the system, without which, health is impossible. Their anti-constipating quality is in the seeds; on the same principle that grapes, raisins, and white mustard-seed, have stood high in this respect, the attrition of the seeds on the mucous surface of the alimentary canal exciting its peristaltic motion, thus causing regular daily action.

As to water-melons, they are the only things we know which can be eaten with impunity, until we cannot swallow any more. The best time for taking them is about eleven o'clock in the morning, and about four in the afternoon. They are not safe for very young children, the seeds are especially injurious to them.

